# Targeting the HDAC6‐Cilium Axis Ameliorates the Pathological Changes Associated with Retinopathy of Prematurity

**DOI:** 10.1002/advs.202105365

**Published:** 2022-05-26

**Authors:** Jie Ran, Yao Zhang, Sai Zhang, Haixia Li, Liang Zhang, Qingchao Li, Juan Qin, Dengwen Li, Lei Sun, Songbo Xie, Xiaomin Zhang, Lin Liu, Min Liu, Jun Zhou

**Affiliations:** ^1^ Institute of Biomedical Sciences Shandong Provincial Key Laboratory of Animal Resistance Biology Collaborative Innovation Center of Cell Biology in Universities of Shandong College of Life Sciences Shandong Normal University Jinan 250014 China; ^2^ State Key Laboratory of Medicinal Chemical Biology College of Life Sciences Haihe Laboratory of Cell Ecosystem Nankai University Tianjin 300071 China; ^3^ Tianjin Key Laboratory of Retinal Functions and Diseases Eye Institute and School of Optometry Tianjin Medical University Eye Hospital Tianjin 300384 China

**Keywords:** cilium, HDAC6, oxygen‐induced retinopathy, photoreceptor, retinopathy of prematurity

## Abstract

Retinopathy of prematurity (ROP) is one of the leading causes of childhood visual impairment and blindness. However, there are still very few effective pharmacological interventions for ROP. Histone deacetylase 6 (HDAC6)‐mediated disassembly of photoreceptor cilia has recently been implicated as an early event in the pathogenesis of ROP. Herein it is shown that enhanced expression of HDAC6 by intravitreal injection of adenoviruses encoding HDAC6 induces the typical pathological changes associated with ROP in mice, including disruption of the membranous disks of photoreceptor outer segments and a decrease in electroretinographic amplitudes. *Hdac6* transgenic mice exhibit similar ROP‐related defects in retinal structures and functions and disassembly of photoreceptor cilia, whereas *Hdac6* knockout mice are resistant to oxygen change‐induced retinal defects. It is further shown that blocking HDAC6‐mediated cilium disassembly by intravitreal injection of small‐molecule compounds protect mice from ROP‐associated retinal defects. The findings indicate that pharmacological targeting of the HDAC6‐cilium axis may represent a promising strategy for the prevention of ROP.

## Graphical abstract

Herein the authors show that overexpression of histone deacetylase 6 (HDAC6) in mice induces the typical pathological changes associated with retinopathy of prematurity (ROP), an eye disease affecting premature infants. Blocking HDAC6‐mediated photoreceptor cilium disassembly protects mice from ROP‐associated retinal defects. This work supports the notion that targeting the HDAC6‐cilium axis holds promise for the prevention of ROP.

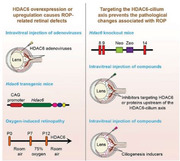

## 1 Introduction

Photoreceptors are highly polarized and compartmentalized neuroepithelial cells in the retina.^[^ ^1^ ^]^ The apical outer segment resembles a highly modified primary cilium, which is connected to the biosynthetic inner segment by a narrow, microtubule‐based connecting cilium analogous to the transition zone of the primary cilium.^[^ ^2^, ^3^ ^]^ The elaborate architecture of the outer segment, also known as the photoreceptor cilium, comprises hundreds of stacked membranous disks and a microtubule‐based ciliary axoneme backbone that extends from the basal body of the inner segment. The membranous disks are highly enriched with proteins essential for phototransduction and renewed through the opposing processes of disk morphogenesis and shedding, which involve a large number of proteins transported through the ciliary axoneme.^[^ ^4^ ^]^ Structural deficits and/or dysfunction of the photoreceptor cilium can cause photoreceptor degeneration and visual impairment, leading to retinal ciliopathies such as retinitis pigmentosa (RP) and Leber congenital amaurosis (LCA).^[^ ^5^, ^6^ ^]^

Our previous study has identified photoreceptor cilium disassembly as an early pathological change associated with retinopathy of prematurity (ROP), adding ROP to the growing list of retinal ciliopathies.^[^ ^7^ ^]^ As a well‐known complication of preterm birth, ROP is initiated by the oxygen supplementation treatment that is necessary to prevent neonatal death and encompasses a spectrum of visual impairments, from a mild disease that resolves spontaneously to severe disease that causes retinal detachment and subsequent blindness. Moreover, even milder forms of ROP can increase the risk of pathologies that adversely affect visual acuity and predispose to strabismus and color discrimination disorders.^[^ ^8^ ^]^ ROP develops following an initial phase of hyperoxia to prevent premature infant death and a subsequent phase of relative hypoxia created by weaning off oxygen supplementation, resulting in pathological neovascularization and subsequent retinal detachment.^[^ ^9^ ^]^ Current therapies to limit the adverse consequences of aberrant neovascularization are invasive or tissue‐destructive, and there are very few effective pharmacological interventions for the specific prevention or treatment of premature infants with ROP.^[^ ^10^, ^11^ ^]^

We have previously revealed that histone deacetylase 6 (HDAC6) is upregulated during the transition from hyperoxia to relative hypoxia.^[^ ^7^ ^]^ HDAC6 is a unique cytoplasmic member of the HDAC family and acts as a key regulator of cilium disassembly, predominantly through its deacetylation of *α*‐tubulin and cortactin.^[^ ^12^ ^]^ Accumulating evidence suggests that HDAC6‐mediated cilium disassembly is involved in the pathogenesis of several ciliopathies, such as chronic obstructive pulmonary disease and cholangiocarcinoma, and small‐molecule compounds inhibiting HDAC6 activity have shown promising therapeutic potential in these diseases.^[^ ^13^, ^14^, ^15^ ^]^ Thus, we hypothesized that HDAC6‐mediated disassembly of the photoreceptor cilium may represent a main cause for ROP and that targeting the HDAC6‐cilium axis may prevent the pathogenesis of this disease. In this study, we demonstrate that HDAC6 overexpression directly triggers photoreceptor cilium disassembly and induces the typical pathological changes associated with ROP, further supporting a pathogenic role for the HDAC6‐cilium axis in ROP. Furthermore, inhibition of the HDAC6‐cilium axis significantly ameliorates ROP‐associated retinal changes, providing a novel avenue for the prevention and treatment of this disease.

## 2 Results

### 2.1 Enhanced Expression of HDAC6 Induces the Typical Pathological Changes Associated with ROP

**Figure 1:**
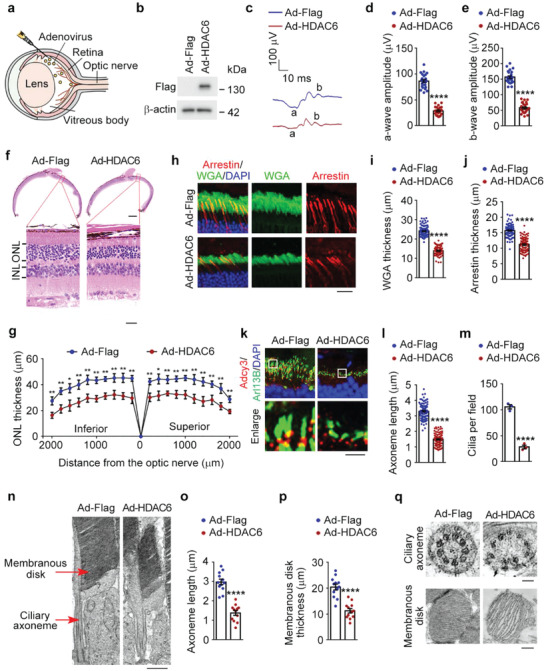
Impairment of photoreceptor structures and functions in mice intravitreally injected with HDAC6 adenoviruses. a) A schematic explanation of intravitreal injection of adenoviruses. Eight‐week‐old mice were intravitreally injected with adenoviruses expressing Flag‐tagged mouse HDAC6 (Ad‐HDAC6) or control adenoviruses (Ad‐Flag). Five days after injection, retinas were extracted and analyzed. b) Immunoblot analysis of Flag and *β*‐actin in retinas of control or HDAC6 adenovirus‐injected mice. c–e) ERG recordings c) and measurement of retinal a‐wave d) and b‐wave e) amplitudes for control and HDAC6 adenovirus‐injected mice under scotopic conditions at 3 cd s m^−2^ flash intensity (*n* = 24 mice from three independent experiments). f,g) Photomicrographs f) and quantification g) of the retinal histology assessed by hematoxylin and eosin (H&E) staining in control and HDAC6 adenovirus‐injected mice (*n* = 12 mice from three independent experiments). ONL, outer nuclear layer. INL, inner nuclear layer. Scale bars, 0.3 mm (top) and 30 µm (bottom). h–j) Immunofluorescence images h) and quantification of the thickness of outer segment membranous disks in rods stained with wheat germ agglutinin (WGA) conjugated with Alexa Fluro 488 i) and cones labeled with the anti‐arrestin antibody j), from control and HDAC6 adenovirus‐injected mice (*n* = 90 fields from 12 mice of three independent experiments). The maximum intensity projection of Z‐stack images was used for quantification of the thickness of membranous disks of rods or cones with the Image J software. Scale bar, 20 µm. k–m) Immunofluorescence images k) and quantification of the length (l, *n* = 90 fields from 12 mice of three independent experiments) of ciliary axonemes and density of cilia (m, n = 3 independent experiments using a total of 12 mice) in retinas stained with antibodies against the ciliary marker Arl13B and the basal body marker Adcy3, from control and HDAC6 adenovirus‐injected mice. The maximum intensity projection of Z‐stack images was used for quantification of ciliary length and density with the Image J software. Scale bar, 3 µm. n–p) Transmission electron microscopy images n) and quantification of the length of ciliary axonemes o), and the thickness of membranous disks p) of the longitudinal sections of photoreceptors in control and HDAC6 adenovirus‐injected mice (*n* = 12 retinas from 6 mice of three independent experiments). Scale bar, 1 µm. q) Transmission electron microscopy images of the cross sections of ciliary axonemes and membranous disks in control and HDAC6 adenovirus‐injected mice. Scale bars, 0.1 µm (top) and 0.5 µm (bottom). Data are presented as mean ± SEM. *p < 0.05, **p < 0.01, ****p < 0.0001.

To gain insights into the direct action of HDAC6 on retinal structures and functions, mice were intravitreally injected with adenoviruses encoding HDAC6 (**Figure** **1**a,b). Eight‐week‐old mice were used for these experiments to mimic ROP patients at the late stage with severe visual impairment. The expression of HDAC6 peaked on the fifth day after adenovirus injection, which showed ≈5‐fold increase compared to the first day (Figure S1a,b, Supporting Information). Thus, mice were analyzed after 5 days of adenovirus injection. The retinal function was examined by full‐field electroretinography (ERG), which reflects the mass electrical response of the retina to photic stimulation.^[^ ^16^ ^]^ We found that the a‐wave of the response, reflecting the general physiological activity of photoreceptors in the outer retina, and the b‐wave reflecting the inner layer activity of ON bipolar cells and Müller cells were both decreased by >50% in HDAC6 adenovirus‐injected mice compared to mice injected with control adenoviruses (Figure 1c–e). Therefore, HDAC6 overexpression in the retina directly induced photoreceptor dysfunction.

The whole retinal histology was then examined by microscopic analysis of hematoxylin and eosin (H&E)‐stained sections. We found that the thickness of the outer nuclear layer (ONL) was reduced by ≈30% in mouse retinas expressing high level of HDAC6, suggesting that HDAC6 overexpression induced photoreceptor abnormalities (Figure 1f,g). To determine the changes in rod and cone photoreceptors, we stained the retina with wheat germ agglutinin (WGA) to label rods,^[^ ^17^ ^]^ and with the anti‐arrestin antibody to label cones.^[^ ^18^ ^]^ Immunofluorescence microscopy revealed that the membranous disks of rods and cones were about 50% thinner in HDAC6 adenovirus‐injected mice than in control mice (Figure 1h–j). Next, we examined the ciliary axoneme, which is critical for the transport of proteins involved in phototransduction into membranous disks. Immunostaining with antibodies targeting Arl13B, a well‐known ciliary marker,^[^ ^19^ ^]^ and Adcy3, a protein localized to the basal bodies of photoreceptor cilia, showed that the intensity and length of the ciliary axoneme were reduced by about 70% and 50%, respectively, in HDAC6 adenovirus‐injected mice (Figure 1k–m). To corroborate these findings, we examined the longitudinal and cross sections of photoreceptors with transmission electron microscopy. Both membranous disks and ciliary axonemes in HDAC6 adenovirus‐injected mice displayed varying degrees of abnormalities (Figure 1n–q). Therefore, intravitreal injection of adenoviruses encoding HDAC6 directly induces the typical pathological changes associated with ROP, including photoreceptor dysfunction and damage of membranous disks and ciliary axonemes.

### 2.2 HDAC6‐Induced Retinal Deficits Recapitulate the Pathological Features of ROP

**Figure 2:**
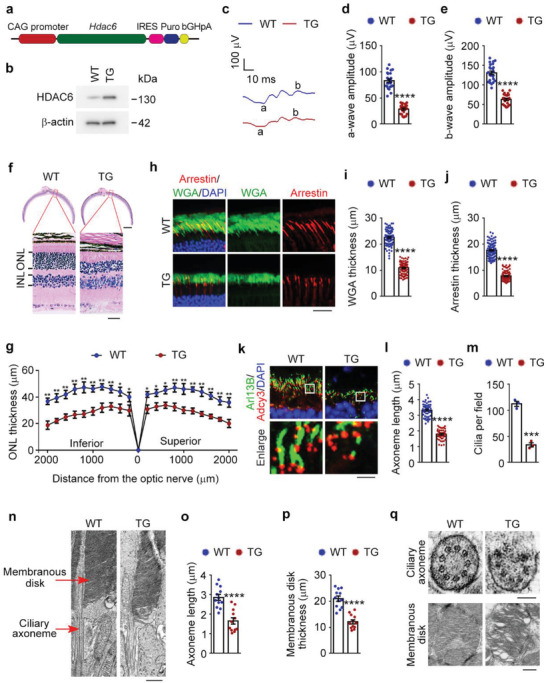
*Hdac6* transgenic mice display the pathological changes associated with ROP. a) An illustration of the overexpression vector used to generate the *Hdac6* transgenic embryonic stem cell line. The vector containing an *Hdac6*‐IRES‐Puro cassette downstream of the chicken *ACBT* (*β*‐actin; CAG) promoter. b) Immunoblot analysis of HDAC6 and *β*‐actin in retinas from 8‐week‐old wild‐type (WT) and *Hdac6* transgenic (TG) mice. c–e) ERG recordings c) and measurement of retinal a‐wave d) and b‐wave e) amplitudes in wild‐type and *Hdac6* transgenic mice under scotopic conditions at 3 cd s m^−2^ flash intensity (*n* = 24 mice from three independent experiments). f,g) Photomicrographs f) and quantification g) of the retinal histology assessed by H&E staining in wild‐type and *Hdac6* transgenic mice (n = 90 fields from 12 mice of three independent experiments). Scale bars, 0.3 mm (top) and 30 µm (bottom). h–j) Immunofluorescence images h) and quantification of the thickness of outer segment membranous disk in rods i) and cones j) from wild‐type and *Hdac6* transgenic mice (*n* = 90 fields from 12 mice of three independent experiments). Scale bar, 20 µm. k–m) Immunofluorescence images k) and quantification of the length (l, *n* = 90 fields from 12 mice of three independent experiments) and density (m, *n* = 3 independent experiments from 12 mice) of ciliary axonemes in retinas from wild‐type and *Hdac6* transgenic mice. Scale bar, 3 µm. n–p) Transmission electron microscopy images n) and quantification of the length of ciliary axonemes o) and the thickness of membranous disks p) of the longitudinal sections of photoreceptors in wild‐type and *Hdac6* transgenic mice (*n* = 12 retinas from 6 mice of three independent experiments). Scale bar, 1 µm. q) Transmission electron microscopy images of the cross sections of ciliary axonemes and membranous disks in wild‐type and *Hdac6* transgenic mice. Scale bars, 0.1 µm (top) and 0.5 µm (bottom). Data are presented as mean ± SEM. *p < 0.05, **p < 0.01, ***p < 0.001, ****p < 0.0001.

To confirm a direct role for the HDAC6‐cilium axis in ROP‐related retinal defects, we used *Hdac6* transgenic mice generated from embryonic stem cells containing the *Hdac6*‐IRES‐*Puro* cassette downstream of the chicken *ACBT* (*β*‐actin) (CAG) promoter (**Figure** **2**a). Transgene incorporation was verified by immunoblotting, which showed an ≈5‐fold increase in the level of HDAC6 in the retina of 8‐week‐old *Hdac6* transgenic mice compared with wild‐type mice (Figure 2b). Then, we examined retinal structures and functions of *Hdac6* transgenic mice. ERG recordings revealed an ≈50% reduction in both a‐wave and b‐wave amplitudes in *Hdac6* transgenic mice compared with wild‐type mice (Figure 2c–e), which is consistent with the adenovirus injection results (Figure 1), indicating the impairment of retinal functions by HDAC6 overexpression.

The retinal morphology was then examined by histopathological analysis, which showed that the ONL thickness was reduced by ≈30% in *Hdac6* transgenic mice compared with wild‐type mice, suggesting that photoreceptors were degenerated in *Hdac6* transgenic mice (Figure 2f,g). We then examined the membranous disks by immunofluorescence microscopy and found that the membranous disks of both rods and cones were reduced by about 50% in *Hdac6* transgenic mice compared with wild‐type mice (Figure 2h–j). *Hdac6* transgenic mice displayed strong defects in retinal photoreceptor cilia (Figure 2k–m). Accordingly, we examined the longitudinal and cross sections of photoreceptors with transmission electron microscopy. We found that the membranous disks and ciliary axonemes of *Hdac6* transgenic mice were disrupted (Figure 2n–q). Collectively, these results demonstrate that transgenic mice overexpressing HDAC6 recapitulate ROP‐related pathological features including deficits in retinal structures and functions.

### 2.3 Mice Subjected to Oxygen Changes Have Structural and Functional Retinal Defects

**Figure 3:**
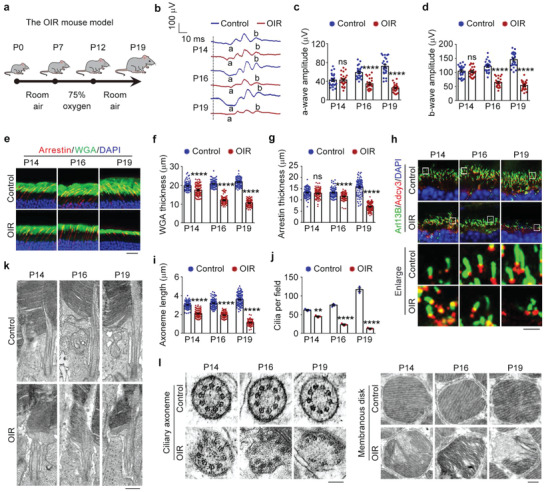
Pathological changes in the OIR mouse model. a) An illustration of the OIR mouse model. The mouse neonates undergo a hyperoxic (75% oxygen) phase from P7 to P12 and a subsequent relative hypoxic phase (room air). b–d) ERG recordings b) and measurement of retinal a‐wave c) and b‐wave d) amplitudes for control and OIR mice under scotopic conditions at 3 cd s m^−2^ flash intensity (*n* = 24 mice from three independent experiments). e–g) Immunofluorescence images e) and quantification of the thickness of outer segment membranous disks in rods f) and cones g) from control and OIR mice (*n* = 90 fields from 12 mice of three independent experiments). Scale bar, 20 µm. h–j) Immunofluorescence images h) and quantification of the length (i, *n* = 90 fields from 12 mice of three independent experiments) and density (j, *n* = 3 independent experiments from 12 mice) of ciliary axonemes in control and OIR mouse retinas. Scale bar, 3 µm. k) Transmission electron microscopy images of the longitudinal sections of photoreceptors in control and OIR mice. Scale bar, 1 µm. l) Transmission electron microscopy images of the cross sections of ciliary axonemes and membranous disks in control and OIR mice. Scale bars, 0.1 µm (left) and 0.5 µm (right). Data are presented as mean ± SEM. **p < 0.01, ****p < 0.0001; ns, not significant.

To investigate whether HDAC6‐mediated cilium disassembly is the main cause of retinal defects in ROP, we exploited the oxygen‐induced retinopathy (OIR) mouse model of human ROP.^[^ ^20^ ^]^ In this model, mouse neonates are subjected to a hyperoxic (75% oxygen) phase from postnatal day 7–12 (P7–P12) and a subsequent relative hypoxia phase created by weaning off oxygen supplementation (**Figure** **3**a), which faithfully mimics the pathogenesis of ROP; in the OIR mouse model, P7 mice are used to mimic the premature infants, and mice older than 12 days correspond to ROP patients at different stages.^[^ ^20^ ^]^ This mouse model adequately reproduces the symptoms of ROP seen in humans and is also useful for assessing treatment outcomes, making it a valuable tool for studying ROP.^[^ ^21^ ^]^ ERG recordings showed that a‐wave and b‐wave amplitudes both decreased in OIR mice starting from P16, indicating photoreceptor dysfunction (Figure 3b–d).

The retinal morphology was then examined by microscopic analysis of H&E‐stained sections, which showed that the ONL thickness was reduced in OIR mice from P16 compared with controls, suggesting that photoreceptors were disturbed in OIR mice (Figure S2a–d, Supporting Information). We next examined the elaborate architectures of photoreceptors, including the light‐sensitive membranous disks and the microtubule‐based ciliary axoneme. Using immunofluorescence microscopy, we found a time‐dependent disruption of the membranous disks of both rods and cones in OIR mice, although the damage in cones (P16) appeared later than in rods (P14) (Figure 3e–g). The intensity and length of the ciliary axoneme also exhibited a time‐dependent damage in the photoreceptors of OIR mice from P14 (Figure 3h–j). Transmission electron microscopy of the longitudinal and cross sections of the membranous disks and ciliary axonemes revealed that their ultrastructures were also damaged in the OIR mice (Figure 3k,l). We also checked the level of HDAC6 and found the upregulation of HDAC6 in the retinas of OIR mice compared with controls (Figure S2e, Supporting Information), consistent with its crucial role in photoreceptor cilium disassembly. To further dissect how oxygen changes impair the integrity of photoreceptors, we investigated whether oxygen changes induce apoptosis of photoreceptors in the OIR mice. Terminal deoxynucleotidyl transferase dUTP nick end labeling (TUNEL) staining demonstrated that the occurrence of apoptosis in photoreceptors was significantly increased from P16 in the OIR mice compared with controls (Figure S2f,g, Supporting Information). Therefore, HDAC6‐mediated photoreceptor cilium disassembly is an early event in response to oxygen changes and is followed by photoreceptor apoptosis and retinal dysfunction, suggesting a crucial role for the HDAC6‐cilium axis in ROP pathology.

### 2.4 Loss of HDAC6 Protects Mice from Oxygen Change‐Induced Retinal Deficits

**Figure 4:**
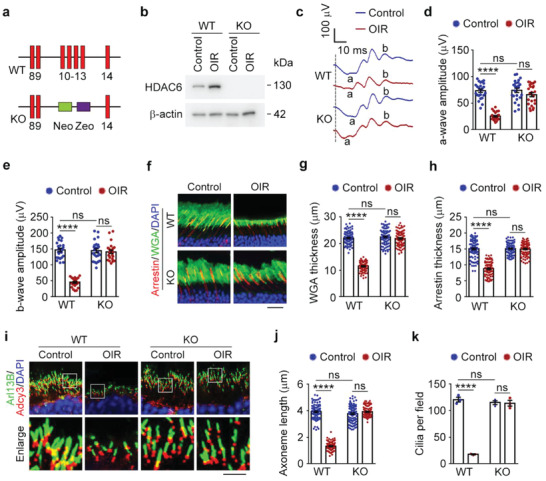
Depletion of HDAC6 protects mice from oxygen change‐induced pathological defects. a) A schematic of the targeting strategy using the mouse *Hdac6* sequence. Exons 10–13 were targeted with a vector containing neomycin (Neo) and zeocin (Zeo) cassettes. b) Immunoblot analysis of HDAC6 and *β*‐actin in retinas from wild‐type and *Hdac6* knockout mice. c–e) ERG recordings c) and measurement of retinal a‐wave d) and b‐wave e) amplitudes for wild‐type (WT) and *Hdac6* knockout (KO) mice (P19) under control or OIR conditions. Stimulus flash at 3 cd s m^−2^ was used to elicit the ERGs under scotopic conditions (*n* = 24 mice from three independent experiments). f–h) Immunofluorescence images f) and quantification of the thickness of outer segment membranous disks of rods g) and cones h) from wild‐type and *Hdac6* knockout mice (P19) under control or OIR conditions (*n* = 90 fields from 12 mice of three independent experiments). Scale bar, 20 µm. i–k) Immunofluorescence images i) and quantification of the length (j, *n* = 90 fields from 12 mice of three independent experiments) and density (k, *n* = 3 independent experiments from 12 mice) of ciliary axonemes in retinas from wild‐type and *Hdac6* knockout mice (P19) under control or OIR conditions. Scale bar, 3 µm. Data are presented as mean ± SEM. ****p < 0.0001; ns, not significant.

Upregulation of HDAC6 could induce the pathological changes associated with ROP, suggesting a critical role for HDAC6 in the pathogenesis of this disease. Thus, we hypothesized that depletion of HDAC6 may prevent ROP‐related retinal defects. To investigate this possibility, we exploited *Hdac6* knockout mice in which exons 10–13 of *Hdac6* were deleted by homologous recombination (**Figure** **4**a). Immunoblotting confirmed the absence of HDAC6 in retinas derived from *Hdac6* knockout mice (Figure 4b).

To explore whether HDAC6 depletion protects against the retinal dysfunction caused by oxygen changes, ERG was performed. Decreases in a‐wave and b‐wave amplitudes due to oxygen changes were significantly abrogated by the loss of HDAC6, even though *Hdac6* knockout mice displayed normal retinal function (Figure 4c–e). We then examined the membranous disks by immunofluorescence analysis. As expected, HDAC6 loss did not affect the membranous disk structure; however, the membranous disk damage induced by oxygen changes was obviously reduced in *Hdac6* knockout mice (Figure 4f–h). Furthermore, the ciliary axoneme defects related to ROP were also ameliorated by HDAC6 depletion, despite *Hdac6* knockout mice exhibiting normal photoreceptor ciliary axonemes (Figure 4i–k). Therefore, loss of HDAC6 protects mice from oxygen change‐induced retinal deficits.

### 2.5 Inhibiting HDAC6 Protects Mice from the Retinal Defects Associated with ROP

Current treatments for ROP mainly target late pathological neovascularization and retinal detachment and have side effects including tissue injury and partial side‐vision loss.^[^ ^10^, ^22^ ^]^ The development of preventative strategies against ROP, especially pharmacological interventions, has been limited by an incomplete understanding of the pathogenesis of this disease. Our findings suggest a potential preventative role for the HDAC6‐cilium axis in ROP, so we sought to investigate whether a small‐molecule HDAC6 inhibitor would prevent the pathological changes associated with ROP. OIR mice were treated with an intravitreal injection of tubastatin A, a selective inhibitor for HDAC6,^[^ ^23^ ^]^ which has been shown to restore cilium assembly in multiple cell lines and organs and used as an investigative treatment for various ciliopathies (Figure S3a, Supporting Information).^[^ ^14^, ^15^, ^24^, ^25^ ^]^

**Figure 5:**
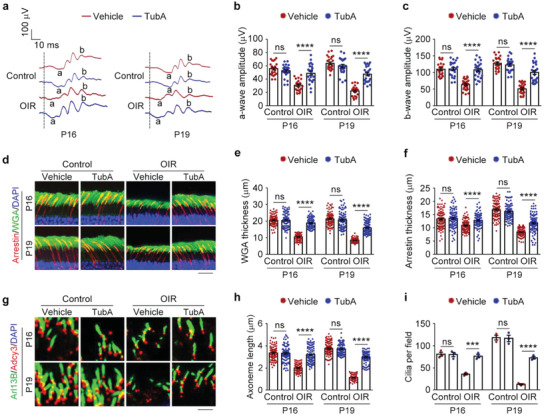
Pharmacological intervention with an HDAC6 inhibitor prevents ROP‐related retinal defects. a–c) ERG recordings a) and measurement of retinal a‐wave b) and b‐wave c) amplitudes in control and OIR mice intravitreally injected with tubastatin A (TubA) or vehicle. Stimulus flash at 3 cd s m^−2^ was used to elicit the ERGs under scotopic conditions (*n* = 24 mice from three independent experiments). d–f) Immunofluorescence images d) and quantification of the thickness of outer segment membranous disks of rods e) and cones f) from control and OIR mice intravitreally injected with tubastatin A or vehicle (*n* = 90 fields from 12 mice of three independent experiments). Scale bar, 20 µm. g–i) Immunofluorescence images g) and quantification of the length (h, *n* = 90 fields from 12 mice of three independent experiments) and density (i, *n* = 3 independent experiments from 12 mice) of ciliary axonemes in retinas from control and OIR mice intravitreally injected with tubastatin A or vehicle. Scale bar, 3 µm. Data are presented as mean ± SEM. ***p < 0.001, ****p < 0.0001; ns, not significant.

We found that intravitreal injection of tubastatin A did not affect retinal structures or functions under normal conditions (**Figure** **5**a–i). However, mice treated with this HDAC6 inhibitor were resistant to oxygen change‐induced retinal dysfunction in ERG recordings (Figure 5a–c) and membranous disk defects (Figure 5d–f). Consistent with numerous previous findings of tubastatin A restoring primary cilia, oxygen change‐related damage to ciliary axonemes of photoreceptors was also significantly reduced by this agent (Figure 5g–i). In addition, analysis of retinal structures and functions for mice at P25 showed that pharmacological inhibition of HDAC6 could prevent the pathological changes associated with ROP until P25 (Figure S3b–j, Supporting Information). These results demonstrate that intravitreal injection of tubastatin A prevents the pathological changes associated with ROP, suggesting that inhibiting HDAC6 may represent a pharmacological preventative strategy for ROP.

### 2.6 Targeting Proteins Upstream of the HDAC6‐Cilium Axis Protects Mice from ROP‐Related Retinal Defects

**Figure 6:**
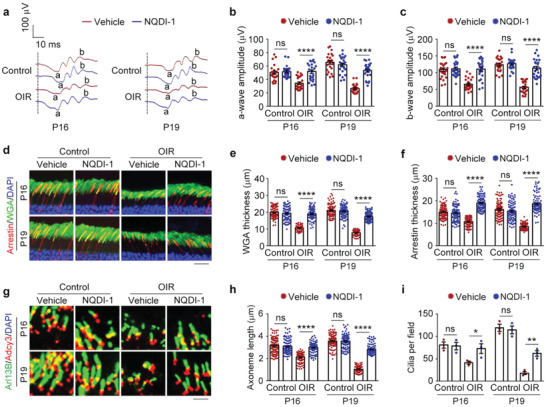
Targeting signaling molecules upstream of HDAC6 blocks the pathological changes associated with ROP. a–c) ERG recordings a) and measurement of retinal a‐wave b) and b‐wave c) amplitudes in control and OIR mice intravitreally injected with NQDI‐1 or vehicle. Stimulus flash at 3 cd s m^−2^ was used to elicit the ERGs under scotopic conditions (*n* = 24 mice from three independent experiments). d–f) Immunofluorescence images d) and quantification of the thickness of outer segment membranous disks of rods e) and cones f) from control and OIR mice intravitreally injected with NQDI‐1 or vehicle (*n* = 90 fields from 12 mice of three independent experiments). Scale bar, 20 µm. g–i) Immunofluorescence images g) and quantification of the length (h, *n* = 90 fields from three independent experiments) and density (i, *n* = 3 independent experiments from 12 mice) of ciliary axonemes in retinas from control and OIR mice intravitreally injected with NQDI‐1 or vehicle. Scale bar, 3 µm. Data are presented as mean ± SEM. *p < 0.05, **p < 0.01, ****p < 0.0001; ns, not significant.

Apoptosis signal‐regulating kinase 1 (ASK1) has been demonstrated to phosphorylate HDAC6 in response to oxygen changes, blocking the ubiquitination and subsequent proteasomal degradation of HDAC6, thus stabilizing this protein.^[^ ^7^ ^]^ Since our work showed the critical involvement of the HDAC6‐cilium axis in the pathogenesis of ROP, we next explored whether targeting ASK1 could ameliorate the pathological changes associated with ROP. NQDI‐1 is a selective inhibitor of ASK1 and has shown great efficacy in several experimental models of human diseases.^[^ ^26^, ^27^, ^28^, ^29^ ^]^ We found that following intravitreal injection of NQDI‐1, the levels of ASK1 phosphorylated at threonine 845 (representing active ASK1) and HDAC6 were both significantly decreased (Figure S4a, Supporting Information). In addition, consistent with the preventative effects of tubastatin A against retinal dysfunction induced by oxygen changes, intravitreal injection of NQDI‐1 also protected mice from ROP‐related retinal dysfunction, as shown by the increased amplitudes of retinal a‐ and b‐waves in OIR mice, although this drug did not obviously affect retinal functions in control mice (**Figure** **6**a–c).

We then performed immunofluorescence microscopy to determine the protective effect of NQDI‐1 on the membranous disks and ciliary axonemes. We found that intravitreal injection of NQDI‐1 did not change the retinal structure in control mice, but could largely alleviated the structural defects caused by oxygen changes (Figure 6d–i). Moreover, the pathological changes related to ROP were also prevented by intravitreal injection of NQDI‐1 until P25 (Figure S4b–j, Supporting Information). Thus, pharmacological inhibition of signaling molecules upstream of HDAC6, such as ASK1, represents another potential strategy for ROP prevention and treatment.

### 2.7 Promoting Photoreceptor Ciliogenesis Protects Mice from the Pathological Changes Associated with ROP

The photoreceptor cilium is essential for light sensation and phototransduction. Structural or functional defects of the photoreceptor cilium could cause photoreceptor degeneration and visual impairment.^[^ ^5^, ^6^ ^]^ Our previous results have demonstrated ROP as a retinal ciliopathy, leading us to determine whether directly promoting photoreceptor ciliogenesis could prevent the pathological changes associated with ROP. OIR mice were treated intravitreally with two different ciliogenesis inducers, cytochalasin D and prostaglandin E2. Cytochalasin D is known to promote ciliogenesis through inhibiting the polymerization of branched F‐actin,^[^ ^12^ ^,^ ^30^ ^]^ and prostaglandin E2 enhances ciliogenesis by modulating intraflagellar transport.^[^ ^31^ ^]^

**Figure 7:**
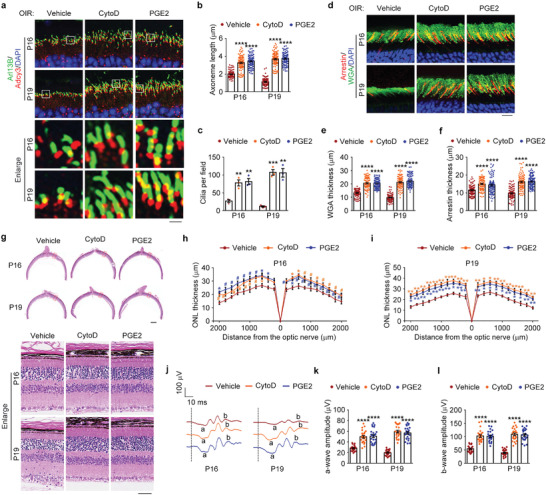
Promoting photoreceptor ciliogenesis ameliorates the pathological changes related to ROP. a–c) Immunofluorescence images a) and quantification of the length (b, *n* = 90 fields from 12 mice of three independent experiments) and density (c, *n* = 3 independent experiments from 12 mice) of ciliary axonemes in retinas from OIR mice intravitreally injected with cytochalasin D (CytoD), prostaglandin E2 (PGE2), or vehicle. Scale bar, 3 µm. d–f) Immunofluorescence images d) and quantification of the thickness of outer segment membranous disks of rods e) and cones f) from OIR mice intravitreally injected with cytochalasin D, prostaglandin E2, or vehicle (*n* = 90 fields from 12 mice of three independent experiments). Scale bar, 10 µm. g–i) Photomicrographs g) and quantification h,i) of the retinal histology assessed by H&E staining in OIR mice intravitreally injected with cytochalasin D, prostaglandin E2, or vehicle (*n* = 3 independent experiments from 12 mice). Scale bars, 0.3 mm (top) and 30 µm (bottom). j–l) ERG recordings j) and measurement of retinal a‐wave k) and b‐wave l) amplitudes in OIR mice intravitreally injected with cytochalasin D, prostaglandin E2, or vehicle. Stimulus flash at 3 cd s m^−2^ was used to elicit the ERGs under scotopic conditions (*n* = 24 mice from three independent experiments). Data are presented as mean ± SEM. *p < 0.05, **p < 0.01, ***p < 0.001 ****p < 0.0001; #, not significant.

We examined whether the ciliary defects induced by oxygen changes could be relieved by these two small‐molecule compounds. Immunofluorescence microscopy of the ciliary axoneme showed that intravitreal injection of cytochalasin D or prostaglandin E2 efficiently blocked the ciliary abnormality in OIR mice (**Figure** **7**a–c). In addition, the membranous disk damage related to ROP was also reduced by these two agents (Figure 7d–f). Because our work demonstrated that abnormalities in ciliary axonemes and membranous disks (P14) could lead to photoreceptor apoptosis and ONL thinning (P16), we evaluated whether directly promoting photoreceptor ciliogenesis could ameliorate these phenotypes. Histopathological analysis showed that the thinner ONL due to oxygen changes was significantly restrained in OIR mice intravitreally injected with cytochalasin D or prostaglandin E2 (Figure 7g–i). Consistent with this finding, the elevated occurrence of photoreceptor apoptosis was also prevented by these two agents (Figure S5a,b, Supporting Information). Furthermore, we checked the retinal function by ERG recordings and found that the decrease in a‐wave and b‐wave amplitudes due to oxygen changes were largely abrogated by cytochalasin D and prostaglandin E2 (Figure 7j–l). These results thus demonstrate that small‐molecule compounds promoting photoreceptor ciliogenesis prevent the pathological changes related to ROP.

## 3 Discussion

Oxygen supplementation is a necessary life‐sustaining measure in premature infants, but it can adversely and permanently affect vision and induce ROP.^[^ ^8^ ^]^ A number of strategies for the prevention or treatment of ROP have been proposed. Current therapeutic approaches mainly focus on the late pathological changes of neovascularization and retinal detachment. Laser photocoagulation has been the mainstay therapy for ROP for many decades; while early interventions in ROP have improved visual outcomes, laser therapy has a long‐term negative impact on visual acuity and fields.^[^ ^22^ ^]^ There are very few clinically approved drugs for the treatment of ROP; drugs targeting vascular endothelial growth factor (VEGF) are used off‐label for ROP, but their application is limited by systemic side effects and a low cost‐benefit ratio.^[^ ^32^, ^33^ ^]^ Preventive measures that target the early pathological changes of ROP have so far shown only modest success, largely because the pathogenesis of this disease is not fully understood.

**Figure 8:**
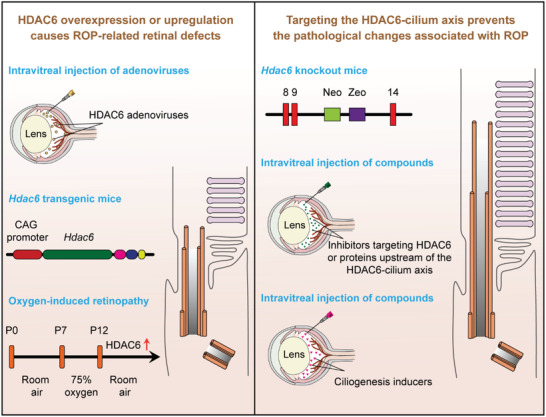
A scheme illustrating the involvement of the HDAC6‐cilium axis in ROP and the potential of targeting this axis in ameliorating retinal defects.

Over the past decade, inhibitors targeting HDACs have shown neuroprotective effects in several diseases of the nervous system, including retinal diseases.^[^ ^34^, ^35^, ^36^ ^]^ Studies using mouse models of inherited blindness demonstrate that pan‐HDAC inhibitors exhibit efficient visual protection.^[^ ^37^, ^38^, ^39^, ^40^ ^]^ However, the clinical application of pan‐HDAC inhibitors has displayed several side effects, such as bone marrow toxicity, thrombocytopenia, fatigue, and diarrhea.^[^ ^41^, ^42^ ^]^ Unlike pan‐HDAC6 inhibitors, selective inhibitors of HDAC6 do not cause obvious toxicity in mice and in clinical trials for the treatment of various diseases.^[^ ^43^, ^44^ ^]^ In several retinal diseases involving oxidative stress, inhibition of HDAC6 shows neuroprotective effects on visual function and retinal morphology. For example, the selective HDAC6 inhibitor tubastatin A has neuroprotective activity for photoreceptors and can restore vision in zebrafish and mouse models of retinal blindness.^[^ ^7^, ^45^, ^46^, ^47^, ^48^ ^]^ Our present work reveals a crucial role for HDAC6‐mediated cilium disassembly in the pathological changes associated with ROP and demonstrates a potential protective effect for targeting the HDAC6‐cilium axis in the prevention of ROP (**Figure** **8**).

The photoreceptor cilium plays an important role in phototransduction. A variety of studies of the structure, function, and molecular components of photoreceptors have highlighted the significance of their ciliary nature.^[^ ^49^, ^50^, ^51^ ^]^ Defects in the photoreceptor cilium have been shown to cause retinal diseases,^[^ ^5^, ^6^ ^]^ and the list of ciliary proteins associated with these retinal diseases is growing. Mutations in over 200 ciliary genes (RetNET, https://sph.uth.edu/retnet/) induce dysfunction and/or death of photoreceptors in retinal diseases, such as RP and LCA, which constitute a significant cause of irreversible vison impairment and vision loss worldwide. For example, *RP1*, a well‐known ciliary gene identified in RP cases, encodes a protein that localizes in the ciliary axoneme of photoreceptors. In mice with *RP1* mutation, the membranous disks are abnormally organized and fail to stack properly, leading to photoreceptor degeneration and retinal dysfunction.^[^ ^52^ ^]^ Centrosome protein 290 (CEP290) localizes in the transition zone (connecting cilium) of the photoreceptor cilium and is required for ciliary trafficking. The photoreceptors of *Cep290*‐depleted mice lack connecting cilia and exhibit the mislocalization of rhodopsin and arrestin and retinal dysfunction.^[^ ^53^ ^]^ HDAC6 is a key regulator of cilium disassembly and exhibits partial localization at the basal body. Our study reveals that overexpression of HDAC6 produces abnormalities in photoreceptor ciliary axonemes and membranous disks, followed by photoreceptor degeneration and dysfunction. Moreover, two ciliogenesis inducers, cytochalasin D and prostaglandin E2, could largely ameliorate the pathological changes associated ROP. Given the crucial role for the cilium in photoreceptor structure and function and the large number of ciliary gene mutations identified in retinal diseases, targeting the photoreceptor cilium and ciliary genes may represent a potential approach for the treatment of retinal ciliopathies.

The HDAC6‐cilium axis is regulated by diverse proteins in various contexts.^[^ ^24^, ^54^, ^55^, ^56^, ^57^, ^58^, ^59^, ^60^ ^]^ Small‐molecule agents targeting this axis have been shown to treat several cilium‐related diseases, such as chronic obstructive pulmonary disease and chondrosarcoma.^[^ ^13^, ^14^, ^15^ ^]^ We have reported that ASK1, an evolutionarily conserved kinase important for stress responses,^[^ ^61^ ^]^ is activated and regulates the HDAC6‐cilium axis in the pathogenesis of ROP.^[^ ^7^ ^]^ Small‐molecule compounds targeting ASK1 have been demonstrated previously to prevent optic nerve degeneration and improve vision in a mouse model of glaucoma.^[^ ^62^ ^]^ Our current results reveal that intravitreal injection of NQDI‐1, a selective ASK1 inhibitor, could largely blocks the pathological changes associated with ROP, suggesting a potential value of targeting ASK1 or other proteins upstream of the HDAC6‐cilium axis for the prevention of ROP. It should be noted, however, that many pharmacological agents display off‐target activity that may cause unexpected adverse effects. Thus, further studies are warranted to investigate the therapeutic potential of these agents.

The past decade has witnessed great advances in the therapy of retinal diseases. For example, gene mutations that cause retinal diseases might be efficiently corrected using genome editing technologies.^[^ ^63^ ^]^ Indeed, genome editing technologies have been applied in the clinic to repair disease‐causing mutations in LCA and RP patients.^[^ ^64^, ^65^ ^]^ In addition, gene therapy using adeno‐associated viruses (AAVs) is a promising strategy for the treatment of retinal diseases, especially photoreceptor degeneration.^[^ ^66^, ^67^ ^]^ In LCA patients with *RPE65* (retinal pigment epithelium 65) mutations, AAVs have been approved by the US Food and Drug Administration and represent one of the first successes in gene therapy of retinal diseases.^[^ ^68^ ^]^ In our studies, *Hdac6* knockout mice did not display any obvious abnormities or pathological changes associated with ROP. It is therefore tempting to speculate that using AAVs to deliver shRNAs targeting *Hdac6* or other components of the HDAC6‐cilium axis might be a promising strategy to prevent ROP.

## 4 Experimental Section

### Animal Use and Care

All animal experiments were conducted according to the Animal Care and Use Committee of Shandong Normal University (AEECSDNU2021048). Animals had free access to food and water and were exposed to normal lighting conditions with 12‐h‐on/12‐h‐off cycles.

### *Hdac6* Transgenic Mice

*Hdac6* transgenic mice were generated and genotyped as described previously.^[^ ^69^ ^]^ In brief, an overexpression vector containing the *Hdac6*‐IRES‐*Puro* cassette downstream of the chicken *ACBT* (*β*‐actin; CAG) promoter was used to construct *Hdac6* transgenic embryonic stem cells, and then *Hdac6* transgenic embryonic stem cells were injected into 4–8‐cell embryos to generate *Hdac6* transgenic mice. Eight‐week‐old mice were used to examine retinal structures and functions.

### *Hdac6* Knockout Mice

*Hdac6* heterozygous mice in the 129/C57BL6 background were produced and genotyped as described previously.^[^ ^70^ ^]^ *Hdac6* heterozygous mice were intercrossed to generate *Hdac6* knockout and wild‐type mice. Both male and female mice were used and sex‐balanced for all experiments.

### OIR Mouse Model

The mouse model of OIR was generated based on a well‐established protocol.^[^ ^20^ ^]^ Briefly, P7 mice were placed in an animal hyperoxia chamber (BioSpherix, Parish, NY) with 75% oxygen for 5 days and then returned to room temperature for additional days until reaching the study endpoint.

### Intravitreal Injection of Adenoviruses and Drugs

Adenoviruses encoding HDAC6 were generated using the pDC315‐Flag vector (GeneChem, Shanghai, China). For adenovirus‐mediated overexpression of HDAC6 in the retina, 8‐week‐old C57BL/6J mice were anaesthetized with 2% isoflurane, and one drop of 0.5% oxybuprocaine hydrochloride (Santen, Osaka, Japan) was applied to the surface of the cornea. The iris was dilated using 0.5% tropicamide phenylephrine (Santen), and mice were intravitreally injected with 1 µL solution containing 4 × 10^10^ PFU mL^−1^ adenoviruses using a 34‐gauge Hamilton needle and syringe (Hamilton Company, Reno, NV) for the following 5 days.

For intravitreal injection of drugs, mouse eyes were treated using the above method. Tubastatin A (sml0044; Sigma‐Aldrich, St Louis, MO) was dissolved in DMSO (0.2 × 10^−3^ m) and diluted 1:10 with 0.9% saline; NQDI‐1 (sml0185; Sigma‐Aldrich) was dissolved in DMSO (1.2 × 10^−3^ m) and diluted 1:10 with 0.9% saline; cytochalasin D (ab143484; Abcam) was dissolved in DMSO (0.1 × 10^−3^ m) and diluted 1:10 with 0.9% saline; prostaglandin E2 (P0409; Sigma‐Aldrich) was dissolved in DMSO (0.5 × 10^−3^ m) and diluted 1:10 with 0.9% saline. Mice were treated by intravitreal injection with 0.5 µL of drugs or the same volume of vehicle every 2 days from P14, when they opened their eyes, until the study endpoint.

### ERG Experiments

Prior to testing, mice were dark‐adapted for 12 h. All handing, preparation, and electrode placements were performed under dim red light to maintain dark adaptation. Mice were anesthetized by intraperitoneal injection with 5% chloral hydrate. The pupils were dilated using one drop of 0.5% tropicamide phenylephrine, and one drop of 0.5% oxybuprocaine hydrochloride was administered for local anesthesia. The body temperature was maintained at 37 °C using a feedback temperature controller (TC‐100, Eaton). The reference electrodes of the visual electrophysiological system (RetiMINER‐C, IRC Technologies) were then placed subcutaneously below the ears. The ground electrode was placed on the tail and the recording electrodes were placed on the corneal surface of each eye. Scotopic ERG responses to white flash stimuli (3 cd s m^−2^) were recorded. The a‐wave amplitude was measured from the baseline to the a‐wave trough and the b‐wave amplitude was measured from the a‐wave trough to the b‐wave peak.

### H&E Staining

Eyeballs were fixed in 10% formalin overnight at 4 °C. The cornea and lens were then removed, and the retinas were fixed for additional 2 h. Retinas were paraffin embedded, and 4 µm retinal sections were prepared. To ensure that sections used for quantification came from the same eccentricity, the eyecups were embedded in the same orientation. Sections encompassing the optic nerve were selected for H&E staining according to the standard protocol. Images were observed and photographed with a DM3000 microscope (Leica, Germany).

### Transmission Electron Microscopy

Mouse eyes were dissected and fixed with 0.25% glutaraldehyde in 0.1 m sodium cacodylate for 4 h at room temperature. After removal of the cornea and lens, the tissues were fixed in 0.25% glutaraldehyde at 4 °C overnight and postfixed in 1% osmium tetroxide for 1 h. After dehydrated through a serious of ethanol, the specimen was infiltrated with resin. The sample was then embedded in Spurr low viscosity resin and cured for 72 h at 65 °C. Finally, the 50 nm ultrathin sections were cut and stained with uranyl acetate and lead citrate as described previously.^[^ ^7^ ^]^ Samples were examined with an HT‐7800 transmission electron microscope (Hitachi) at 80 kV.

### Immunofluorescence Staining

For staining of membranous disks, anesthetized mice were transcardially perfused with fixative solution containing 4% paraformaldehyde with PIPES (80 × 10^−3^ m, pH 6.8), EGTA (5 × 10^−3^ m), and MgCl_2_ (2 × 10^−3^ m). Eyes were enucleated and postfixed in the same fixative solution overnight at 4 °C. After fixation, the eyes were dissected, and eyecups were embedded in 5% low‐melt agarose (A600015‐0025; Sangon Biotech, Shanghai, China) and cut with a vibratome (VT1200S; Leica) into 80 µm slices. The sections were blocked in solutions containing 7% goat serum and 0.5% Triton X‐100 for 30 min at room temperature. Then, slices were incubated with WGA (1 µg mL^−1^; W11261; Thermo Fisher Scientific, Waltham, MA) conjugated with Alexa Fluro 488 (A21202; Thermo Fisher Scientific) and anti‐arrestin antibodies (ab15282; Millipore, Burlington, MA) at 4 °C overnight. The sections were washed three times in phosphate‐buffered saline (PBS) and incubated with Alexa Fluro 568 (A10042; Thermo Fisher Scientific) at room temperature for 1 h. Slides were then washed in PBS three times and stained with DAPI. After sections were washed another three times in PBS and mounted onto slides in glycerol, images were taken using the Dragonfly confocal imaging system (Dragonfly200; Andor Technology, Belfast, UK).

For staining of the ciliary axonemes of photoreceptors, the entire eyes from euthanized mice were extracted and fixed with 4% paraformaldehyde for 30 s at room temperature, and after cryo‐embedding in Tissue‐Tek OCT (Sakura), 10 µm frozen sections were cut using a freezing microtome (CM3050S; Leica). Frozen sections were incubated with anti‐Arl13B (ab136648; Abcam) and anti‐Adcy3 (19492‐1‐AP; Proteintech) antibodies at 4 °C overnight. After washing in PBS three times, slides were incubated with Alexa Fluro 488 (A21202; Thermo Fisher Scientific) and Alexa Fluro 568 (A10042; Thermo Fisher Scientific) for 1 h at room temperature. Slides were then washed in PBS three times and stained with DAPI. For immunofluorescence staining of retinas from adenoviruses‐injected mice, the entire eyes were extracted and fixed with 4% paraformaldehyde at 4 °C overnight. After cryo‐embedding, the 10 µm frozen sections were cut as described above and incubated with the anti‐Flag antibody (F1084; Sigma‐Aldrich) at 4 °C overnight. The slides were then stained with Alexa Fluro 488 (A21202; Thermo Fisher Scientific) and DAPI as described above. TUNEL staining was performed using an in situ cell death detection kit (11 684 795 910; Roche) according to the manufacturer's instructions, and the slides were counterstained with DAPI. Images were taken using a Lecia SP8 confocal microscope and analyzed with the 3D‐analysis tool of the Leica Application Suite X software.

### Immunoblotting

Whole retinas were extracted from mice after removal of the lens and cornea and then lysed using a tissue lyser (JXFSTPRP‐24L; Jingxin, Shanghai, China), in cold lysis buffer containing Tris (50 × 10^−3^ m, PH 7.5), NaCl (150 × 10^−3^ m), EDTA (1 × 10^−3^ m), glycerin (3%), NP40 (1%), and complete protease inhibitor tablets (Roche, Basel, Switzerland). Lysates were cleared by centrifugation at 15 000 rpm for 20 min at 4 °C. Supernatants were added with SDS sample buffer (pH 6.8) containing urea (8 m) and subjected to 8% SDS‐PAGE. Proteins were transferred to polyvinylidene difluoride membranes (Millipore) and subjected to immunoblot analysis as described previously.^[^ ^7^ ^]^ Primary antibodies for immunoblotting were as follows: HDAC6 (07‐732; Millipore), pT845‐ASK1 (3765; Cell Signaling Technology), *α*‐tubulin (ab18251; Abcam), acetylated‐*α*‐tubulin (T6793; Sigma‐Aldrich), and *β*‐actin (A5316, Sigma‐Aldrich).

### Statistical Analysis

The GraphPad Prism 8.0 software (GraphPad software, La Jolla, CA) was used for statistical analysis. Experimental data are presented as mean ± SEM. For normally distributed data, student's *t*‐test was performed to evaluate differences between two sets, and two‐way analysis of variance (ANOVA) was employed to compare three or more sets. For data not normally distributed, nonparametric statistics was performed. A *p*‐value < 0.05 was considered statistically significant.

## Conflict of Interest

The authors declare no conflict of interest.

## Supporting information

Supporting Information

## Data Availability

The data that support the findings of this study are available from the corresponding author upon reasonable request.
