# Supplementary material for: Targeting the HDAC6‐Cilium Axis Ameliorates the Pathological Changes Associated with Retinopathy of Prematurity
Source: Adv Sci (Weinh). 2022 May 26;9(21):2105365. doi: 10.1002/advs.202105365 (PMC9313505; doi:10.1002/advs.202105365)
Supplement: Supplementary file 1 — Supporting Information [file ADVS-9-2105365-s001.pdf]

Supporting Information

Targeting the HDAC6-cilium axis ameliorates the pathological changes associated with retinopathy of prematurity

Jie Ran[1], Yao Zhang[1], Sai Zhang[1], Haixia Li[1], Liang Zhang[1], Qingchao Li[1], Juan Qin[2], Dengwen Li[2], Lei Sun[1], Songbo Xie[1], Xiaomin Zhang[3], Lin Liu[2], Min Liu[1], and Jun Zhou[1,2]*

[1]Institute of Biomedical Sciences, Shandong Provincial Key Laboratory of Animal Resistance Biology, Collaborative Innovation Center of Cell Biology in Universities of Shandong, College of Life Sciences, Shandong Normal University, Jinan 250014, China.
[2]State Key Laboratory of Medicinal Chemical Biology, College of Life Sciences, Haihe Laboratory of Cell Ecosystem, Nankai University, Tianjin 300071, China.
[3]Tianjin Key Laboratory of Retinal Functions and Diseases, Eye Institute and School of Optometry, Tianjin Medical University Eye Hospital, Tianjin 300384, China.

*Correspondence: junzhou@sdnu.edu.cn (J.Z.)

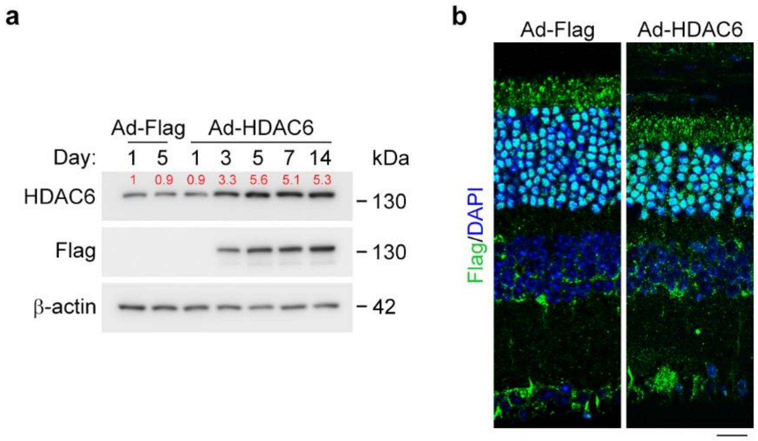

Figure S1. Adenovirus-mediated HDAC6 overexpression in mouse retinas.

a) Immunoblot analysis of HDAC6 and β-actin in the retinas of mice intravitreally injected with adenoviruses expressing Flag-tagged HDAC6 (Ad-HDAC6) or control adenoviruses (Ad-Flag).

b) Immunofluorescence images of retinas of control or HDAC6 adenovirus-injected mice. Scale bar, 10 μm.

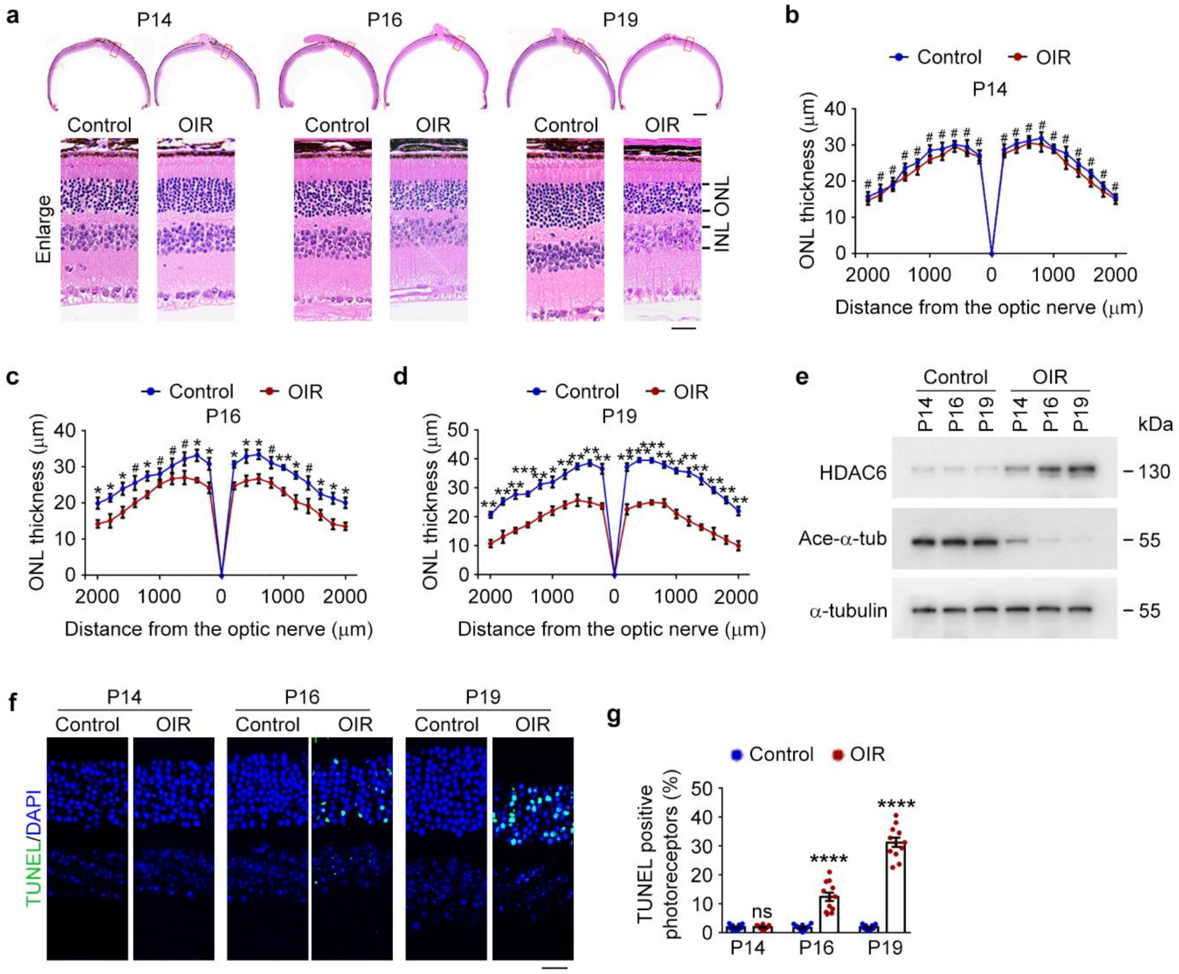

Figure S2. Photoreceptor apoptosis occurs in the late stage in the OIR mouse model.

a-d) Photomicrographs (a) and quantification (b-d) of the retinal histology assessed by H&E staining in control and OIR mice (n = 3 independent experiments). ONL, outer nuclear layer. INL, inner nuclear layer. Scale bars, 0.3 mm (top) and 30 μm (bottom).

e) Immunoblot analysis of HDAC6, acetylated α-tubulin (ace-α-tub), and α-tubulin in the retinas of control and OIR mice.

f, g) Fluorescence images (f) and quantification (g) of TUNEL stained cells in control and OIR retinas. Scale bar, 10 μm.

Data are presented as mean ± SEM. *p < 0.05, **p < 0.01, ***p < 0.001 ****p < 0.0001; ns or #, not significant.

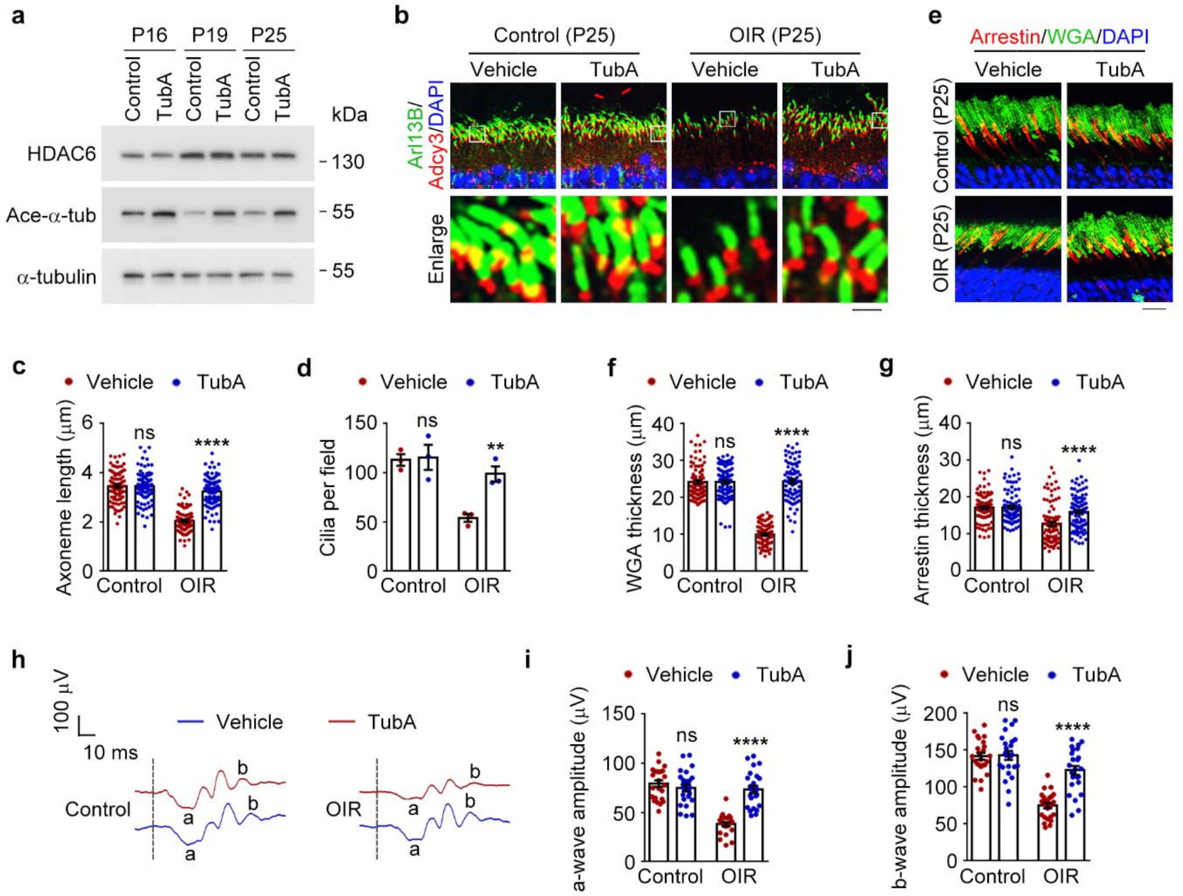

Figure S3. Intravitreal injection of tubastatin A prevents the pathological changes associated with ROP.

a) Immunoblot analysis of HDAC6, acetylated α-tubulin (ace-α-tub), and α-tubulin in the retinas of OIR mice intravitreally injected with tubastatin A (TubA) or vehicle.

b-d) Immunofluorescence images (b) and quantification of the length (c, n = 90 fields from three independent experiments) and density (d, n = 3 independent experiments) of ciliary axonemes in retinas from control and OIR mice intravitreally injected with tubastatin A or vehicle.

e-g) Immunofluorescence images (e) and quantification of the thickness of outer segment membranous disks of rods (f) and cones (g) from control and OIR mice intravitreally injected with tubastatin A or vehicle (n = 90 fields from three independent experiments). Scale bar, 10 μm.

h-j) ERG recordings (h) and measurement of retinal a-wave (i) and b-wave (j) amplitudes in control and OIR mice intravitreally injected with tubastatin A or vehicle. Stimulus flash at 3 cd·s/m^2 was used to elicit the ERGs under scotopic conditions (n = 24 mice from three independent experiments).

Data are presented as mean ± SEM. **p < 0.01, ****p < 0.0001; ns, not significant.

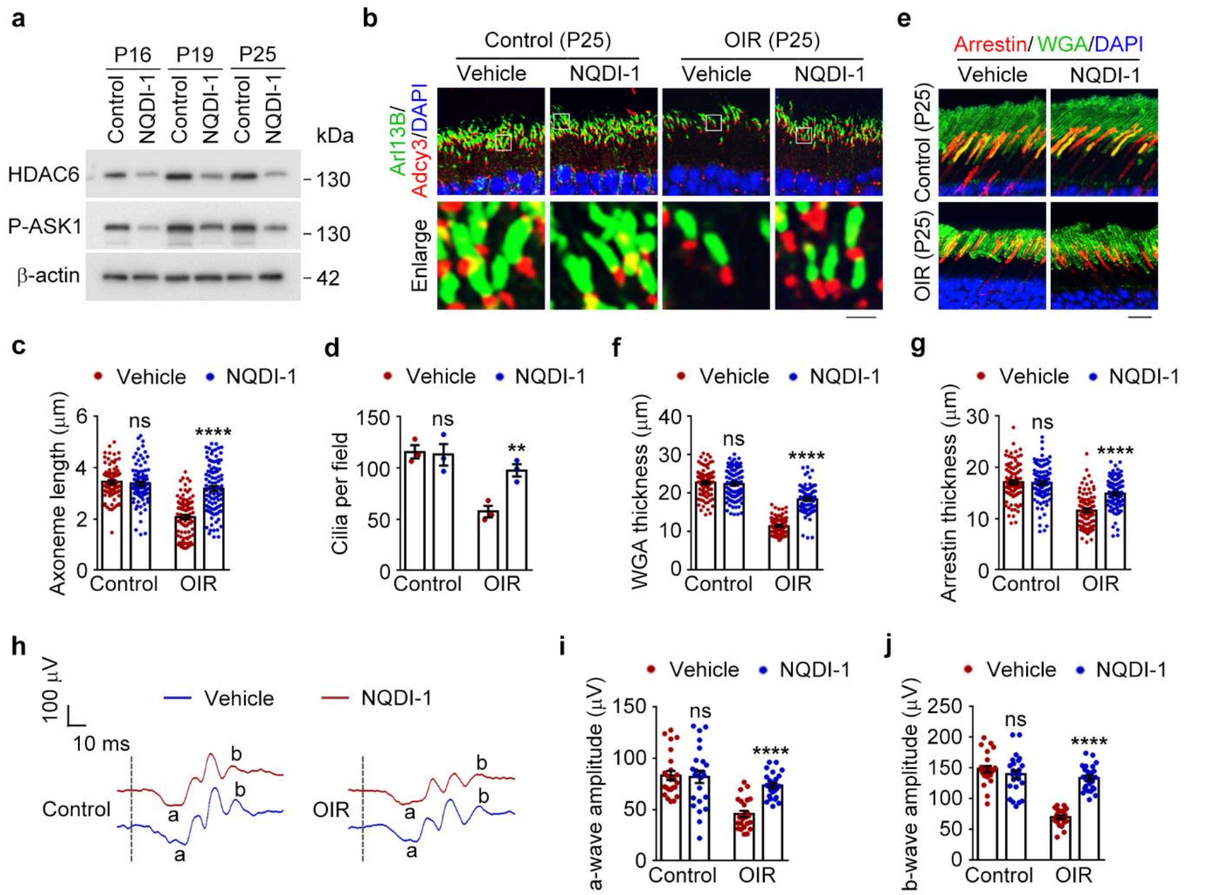

Figure S4. Intravitreal injection of NQDI-1 blocks the pathological changes associated with ROP.

a) Immunoblot analysis of HDAC6, ASK1 phosphorylated at threonine 845 (P-ASK1), and β-actin in retinas from OIR mice intravitreally injected with NQDI-1 or vehicle.

b-d) Immunofluorescence images (b) and quantification of the length (c, n = 90 fields from three independent experiments) and density (d, n = 3 independent experiments) of ciliary axonemes in retinas from control and OIR mice intravitreally injected with NQDI-1 or vehicle.

e-g) Immunofluorescence images (e) and quantification of the thickness of outer segment membranous disks of rods (f) and cones (g) from control and OIR mice intravitreally injected with NQDI-1 or vehicle (n = 90 fields from three independent experiments). Scale bar, 10 μm.

h-j) ERG recordings (h) and measurement of retinal a-wave (i) and b-wave (j) amplitudes in control and OIR mice intravitreally injected with NQDI-1 or vehicle. Stimulus flash at 3 cd·s/m^2 was used to elicit the ERGs under scotopic conditions (n = 24 mice from three independent experiments).

Data are presented as mean ± SEM. **$p < 0.01$, ****$p < 0.0001$; ns, not significant.

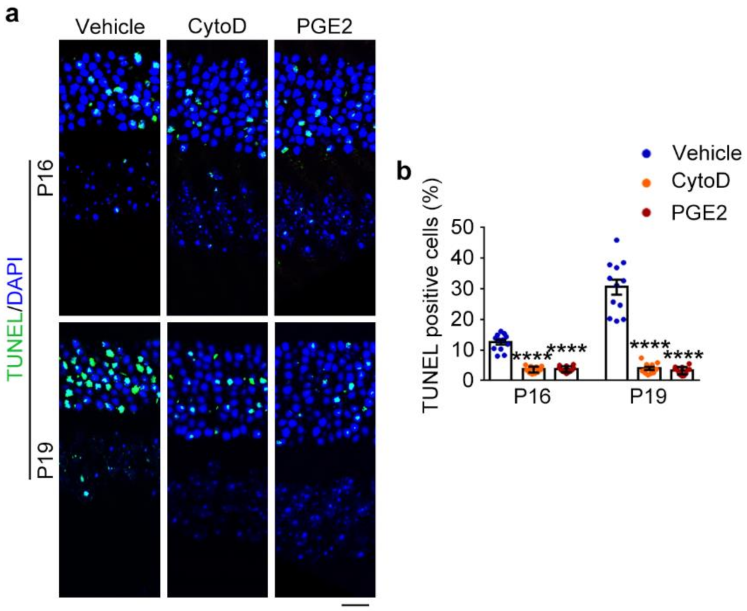

Figure S5. Promoting ciliogenesis restrains photoreceptor apoptosis in response to oxygen changes.

a, b) Fluorescence images (a) and quantification (b) of TUNEL-stained cells in the retinas of OIR mice intravitreally injected with cytochalasin D (CytoD), prostaglandin E2 (PGE2), or vehicle. Scale bar, 10 μm.

Data are presented as mean ± SEM. ****$p < 0.0001$.
